# Supplementary material for: Phosphorylation by Aurora B kinase regulates caspase-2 activity and function
Source: Cell Death Differ. 2020 Aug 18;28(1):349–66. doi: 10.1038/s41418-020-00604-y (PMC7852673; doi:10.1038/s41418-020-00604-y)
Supplement: Supplementary file 1 — Supplemental Table 1 [file 41418_2020_604_MOESM1_ESM.docx]

| **Amino acid** | **Mutation** | **Forward primer (5’ > 3’)** | **Reverse primer (5’ > 3’)** |
| --- | --- | --- | --- |
| **S24** | A - GCC | CAGGAGGGCCAGGATTTTGG | CCAAAATCCTGGCCCTCCTG |
|  | E - GAG | CAGGAGGGAGAGGATTTTGG | CCAAAATCCTCTCCCTCCTG |
| **S80** | A - GCT | CAAAGGGGGCGCTTTCAGCC | GGCTGAAAGCGCCCCCTTTG |
|  | E - GAG | CAAAGGGGGCGAGTTCAGCC | GGCTGAACTCGCCCCCTTTG |
| **T158** | V - GTA | CCTATCCGTAGATGCTACGG | CCGTAGCATCTACGGATAGG |
|  | E - GAG | CCTATCCGAGGATGCTACGG | CCGTAGCATCCTCGGATAGG |
| **T161** | V - GTG | CAGATGCTGTGGAACACTCC | GGAGTGTTCCACAGCATCTG |
|  | E - GAG | CAGATGCTGAGGAACACT CC | GGAGTGTTCCTCAGCATCTG |
| **T180** | V - GTT | GCCATGCGTTCCTGAGTTTTAC | GTAAAACTCAGGAACGCATGGC |
|  | E - GAG | GCCATGCGAGCCTGAGTTTTAC | GTAAAACTCAGGCTCGCATGGC |
| **S220** | A - GCT | GAATTCCGCGCTGGAGGGGATG | CATCCCCTCC AGCGCGGAATTC |
|  | E - GAG | GAATTCCGCGAGGGAGGGGATG | CATCCCCTCC CTCGCGGAATTC |
| **S346** | A - GCT | GTGAGGAGGCTGATGCTGGC | GCCAGCATCAGCCTCCTCAC |
|  | E - GAG | GTGAGGAGGAGGATGCTGGC | GCCAGCATCCTCCTCCTCAC |
| **S384** | A - GCC | CAAACGGGGTGCCTGGTACATTG | CAATGTACCAGGCACCCCGTTTG |
|  | G - GGC | CAAACGGGGTGGCTGGTACATTG | CAATGTACCAGCCACCCCGTTTG |
|  | T - ACC | CAAACGGGGTACCTGGTACATTG | CAATGTACCAGGTACCCCGTTTG |
|  | E - GAG | CAAACGGGGTGAGTGGTACATTG | CAATGTACCACTCACCCCGTTTG |

**Supplementary Table S1. Primers sequences used for mutagenesis.** A, alanine; T, threonine; S, serine; E, glutamate; G, glycine; V, valine.
