# Supplementary material for: Phosphorylation by Aurora B kinase regulates caspase-2 activity and function
Source: Cell Death Differ. 2020 Aug 18;28(1):349–66. doi: 10.1038/s41418-020-00604-y (PMC7852673; doi:10.1038/s41418-020-00604-y)
Supplement: Supplementary file 2 — Supplemental Figure Legends [file 41418_2020_604_MOESM2_ESM.docx]

**Supplementary Figure legends**

**Supplementary Figure S1. Purification of mouse caspase-2-GFP.** Recombinant mouse caspase-2-C320G-GFP was expressed and purified as described in Materials and Methods. Representative Coomassie blue stained gel showing purified mouse full length (fl) caspase-2-C320G-GFP (Casp2-GFP) in ‘Elution 1’.

**Supplementary Figure S2. MS/MS-based evidence for caspase-2 phosphorylation.** The MS/MS spectra illustrating the identification of peptides containing phosphorylated Ser24, Ser80, Ser157, Thr158, Thr161, Ser164, Thr180, Ser220, Ser340, Ser346 and Ser384 in mouse caspase-2. Fragment ions (y ions =red ticks; b ions = blue ticks).

**Supplementary Figure S3. Transient expression of caspase-2-S384E mutant shows reduced cell death.** For cell death assays, *Casp2^-/-^* immortalised MEFs were co-transfected with GFP-caspase-2 WT, C320G, S384A or S384E and β-gal reporter plasmids. After 24 h, cells were fixed and incubated with an X-gal containing solution. Blue (transfected) cells were counted for apoptotic morphology under microscope.  **a.** Representative images showing X-gal staining with live or apoptotic cell morphology. Arrow, dead cell; arrowhead, live cell**.** Scale bar=50μm **b**. Graph showing % cell death, quantitated from ~300-400 β-gal positive cells per experiment (n=3-4 independent experiments). ****, p<0.0001 compared to WT; ^####^, p<0.0001 compared to S384A; mean ± SEM. One-way ANOVA with post hoc test.

**Supplementary Figure S4. Caspase-2-S384E mutation affects catalytic activity. a.** GFP mock vector, GFP-caspase-2 WT, C320G or phosphorylation site mutants were transiently expressed in U2OS-*CASP2^-/-^* cells. The cell lysates were subjected to caspase activity using VDVAD-AFC as a substrate, as described in Materials and Methods. mean ± SEM; n=4; *, p<0.05; **, p<0.01; ***, p<0.001; ****, p<0.0001 compared to GFP-caspase-2 WT. **b.** GFP-caspase-2 WT, C320G or various S384 mutants, S384A, S384E, S384G and S384T, were transiently expressed in U2OS-*CASP2^-/-^* cells for 24 h. Protein lysates were subjected to immunoblotting with the indicated antibodies. Stain-free membrane was used as loading control. NT, non-transfected, fl,full length; cl, cleaved.

**Supplementary Figure S5. Aurora A kinase does not phosphorylate the S384 residue in caspase-2 *in vitro.*** GST-Casp2-C320G (S384 WT), GST-Casp2-C320G-S384A (S384A) or GST was subjected to *in vitro* phosphorylation by incubation with AURKA and [γ-^32^P]-ATP. Autoradiography analysis shows AURKA phosphorylation of both full length (fl) WT and S384A mutant. IB with GST antibody shows GST-tagged Casp2 protein loading. MBP, myeloid basic protein was used as positive control for AURKA phosphorylation.

**Supplementary Figure S6. Immuno-localisation of GFP-caspase-2-C320G and S384E proteins.** Stable cell lines were generated using U2OS or U2OS-*CASP2^-/-^* cells as described in Materials and Methods. U2OS cells expressing GFP (sWT) or U2OS-*CASP2^-/-^* cells expressing GFP (sKO), C320G (sC320G) or S384E (cell clones sS384E #1 and S384E #2) were stained with GFP antibody (red) and DAPI (blue) as described in Materials and Methods. Images were taken with a confocal microscope, LSM700. Scale bar=20μm.

**Supplementary Figure S7. Cytokinesis inhibition results in increased polyploidy in U2OS-*CASP2^-/-^* cells.** U2OS parental or *CASP2^-/-^* cells were treated with DMSO, aurora kinase B inhibitor, AZD1152-HQPA (AZD, 400nM) or the myosin-II inhibitor blebbistatin (Bleb, 50 µM) for 0 h, 24 h and 48 h, followed by immunoblot and DNA content analysis. **a.** Representative immunoblots of cell lysates from treated U2OS parental and *CASP2^-/-^* cells. Antibodies used for immunoblotting are as indicated. β-actin was used as loading control. fl, full length; cl, cleaved **b.** Flow cytometric profiles of the DNA content in U2OS parental and U2OS-*CASP2^-/-^* cells following AZD or Bleb treatment. The percentage represents polyploid cells (> 4N) which was calculated using FACS Express 6 cell cycle analysis module.

**Supplementary Figure S8. Cleavage of Mdm2 is rescued by re-expression of Caspase-2-WT and S384A in *CASP2^-/-^* cells.** U2OS, U2OS-*CASP2^-/-^* , A549 and A549-*CASP2^-/-^* cells were transfected with 200 ng (for U2OS and U2OS-*CASP2^-/-^* cells) or 500 ng (for A549 and A549-*CASP2^-/-^* cells) of GFP or GFP-caspase-2 WT, C320G, S384A or S384E for 6 h, followed by treating with DMSO or 2 µM ZM447439 (ZM) for 24 h. The cell lysates were subjected to immunoblot. **a** and **b.** Representative immunoblots (from three independent experiments) of cell lysates from treated U2OS and U2OS-*CASP2^-/-^* cells (**a**) or A549 and A549-*CASP2^-/-^* cells (**b**). Antibodies used for immunoblotting are as indicated. Stain-free membrane was used as loading control.endo, endogenous; fl, full length; cl, cleaved.

**Supplementary Figure S9. Cleavage of caspase-2 C320G and S384E in U2OS cells is mediated by caspase-3. a and b.** U2OS-*CASP2*^-/-^ or U2OS-*CASP2*^-/-^/*3*^-/-^ cells were left untransfected (NT) or were transfected with GFP mock vector, GFP-caspase-2 WT, C320G or S384E. Cell lysates were subjected to immunoblotting with the indicated antibodies. β-actin was used as loading control. fl, full length.
