# Supplementary figures and images for: Phosphorylation by Aurora B kinase regulates caspase-2 activity and function
Source: Cell Death Differ. 2020 Aug 18;28(1):349–66. doi: 10.1038/s41418-020-00604-y (PMC7852673; doi:10.1038/s41418-020-00604-y)

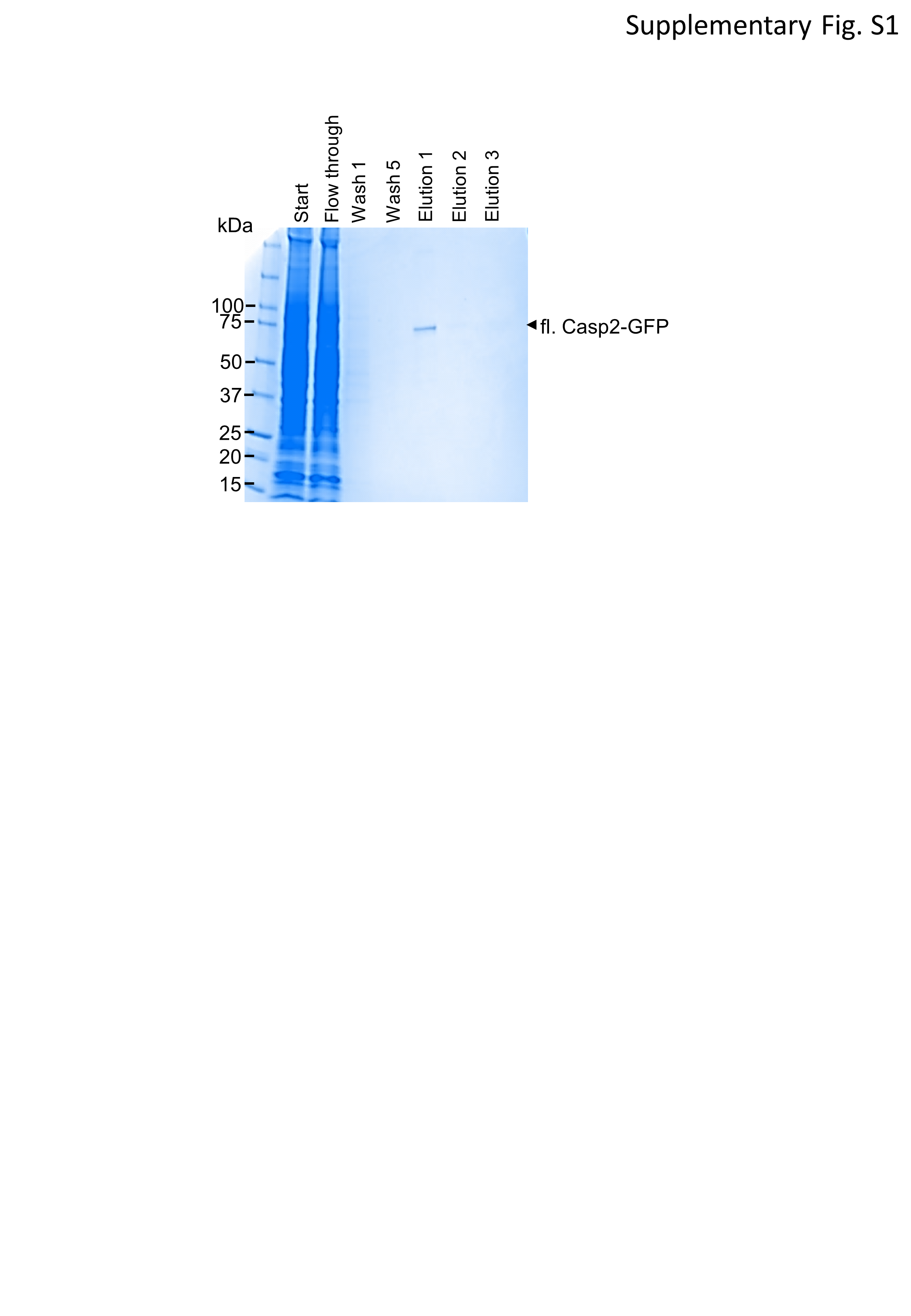

Supplement: Supplementary file 3 — Supplemental Figure 1 [file 41418_2020_604_MOESM3_ESM.tif]

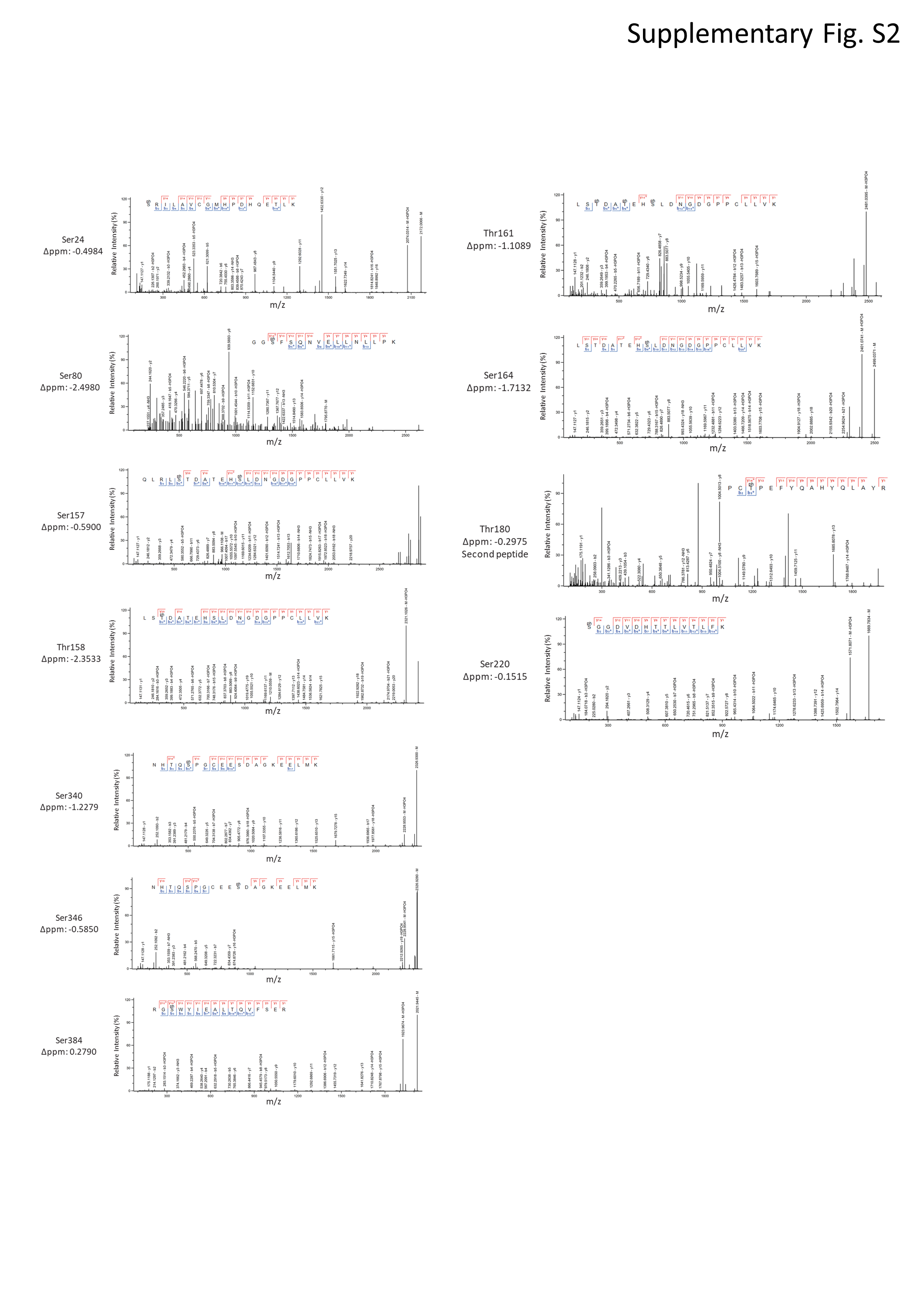

Supplement: Supplementary file 4 — Supplemental Figure 2 [file 41418_2020_604_MOESM4_ESM.tif]

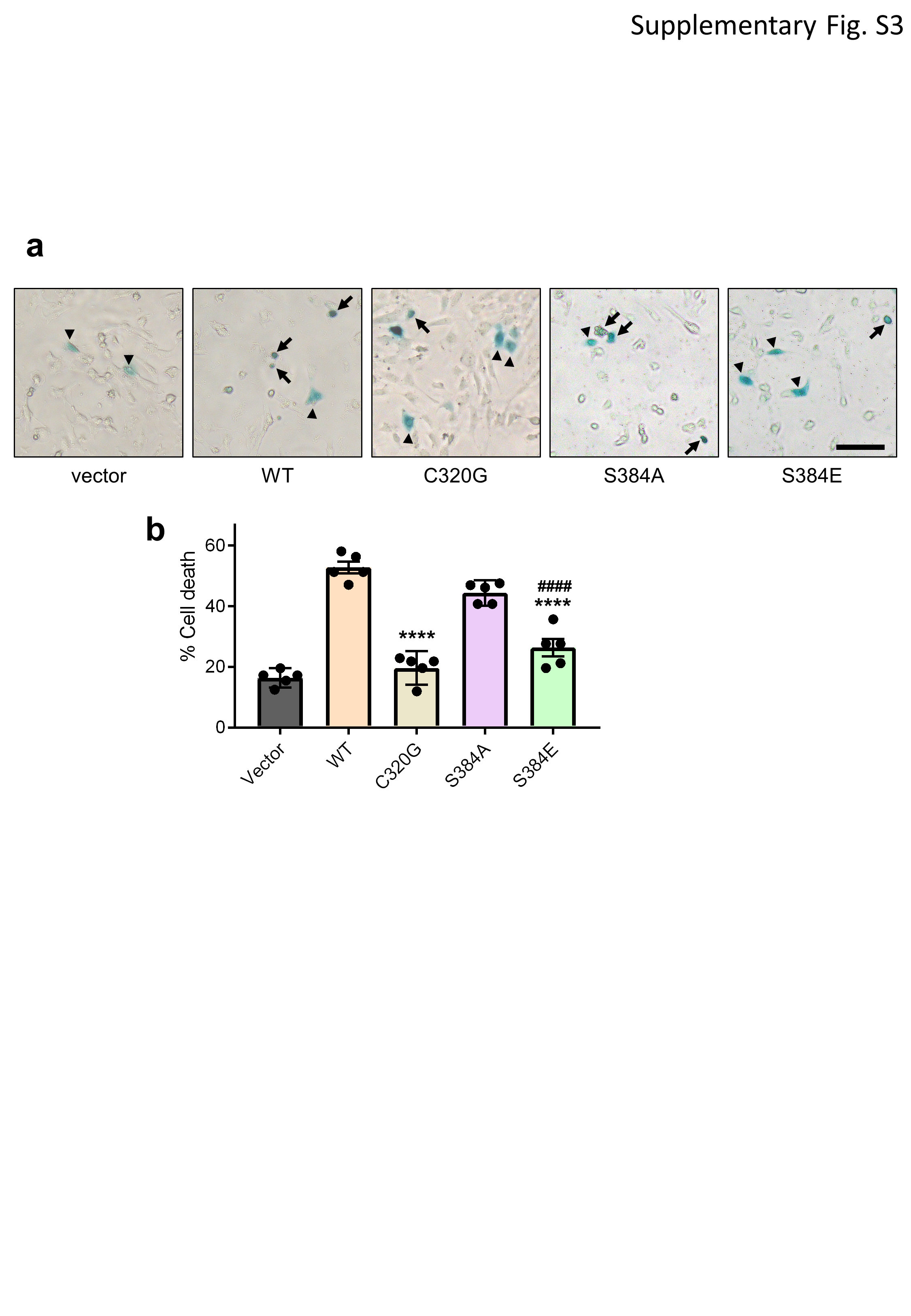

Supplement: Supplementary file 5 — Supplemental Figure 3 [file 41418_2020_604_MOESM5_ESM.tif]

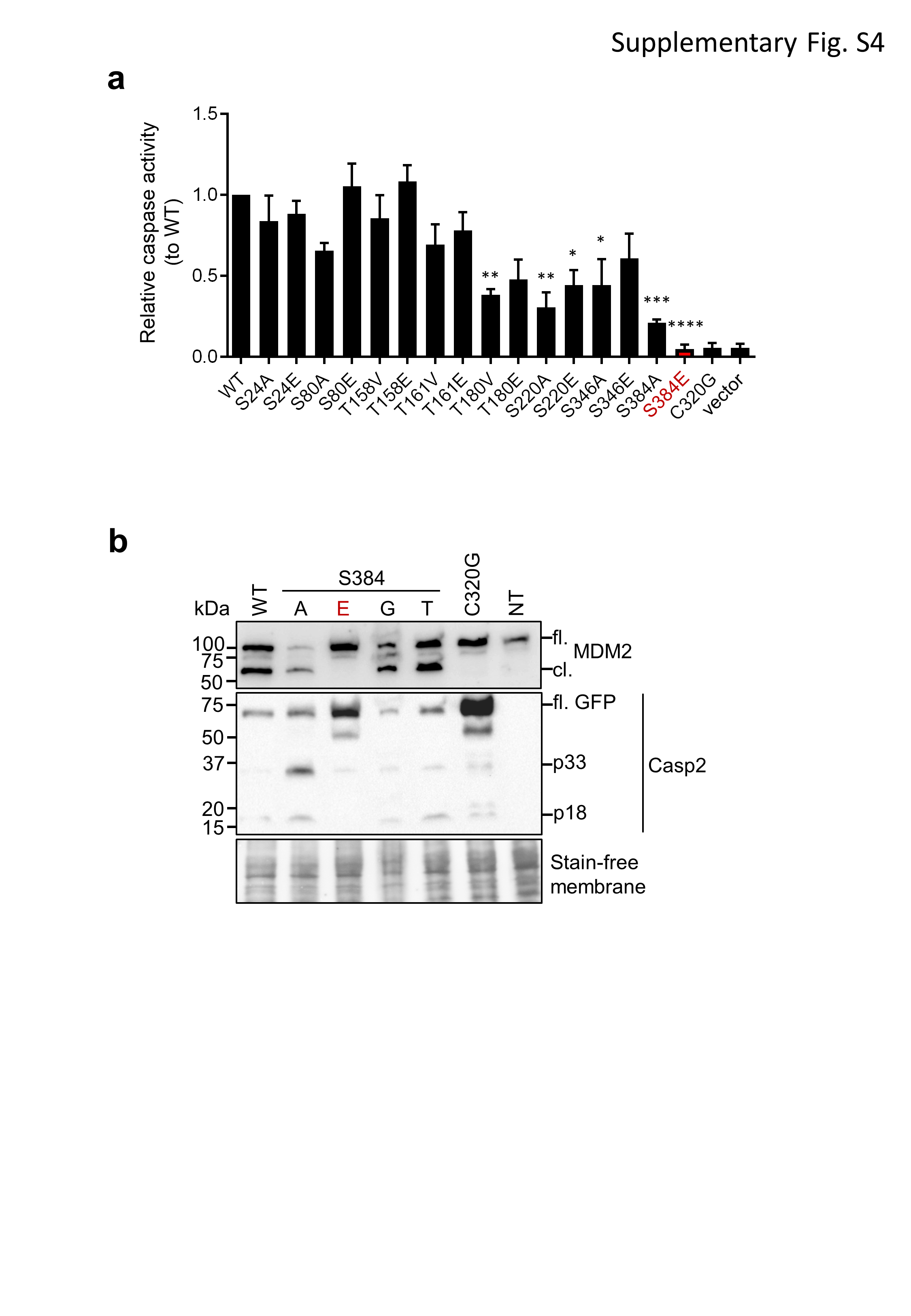

Supplement: Supplementary file 6 — Supplemental Figure 4 [file 41418_2020_604_MOESM6_ESM.tif]

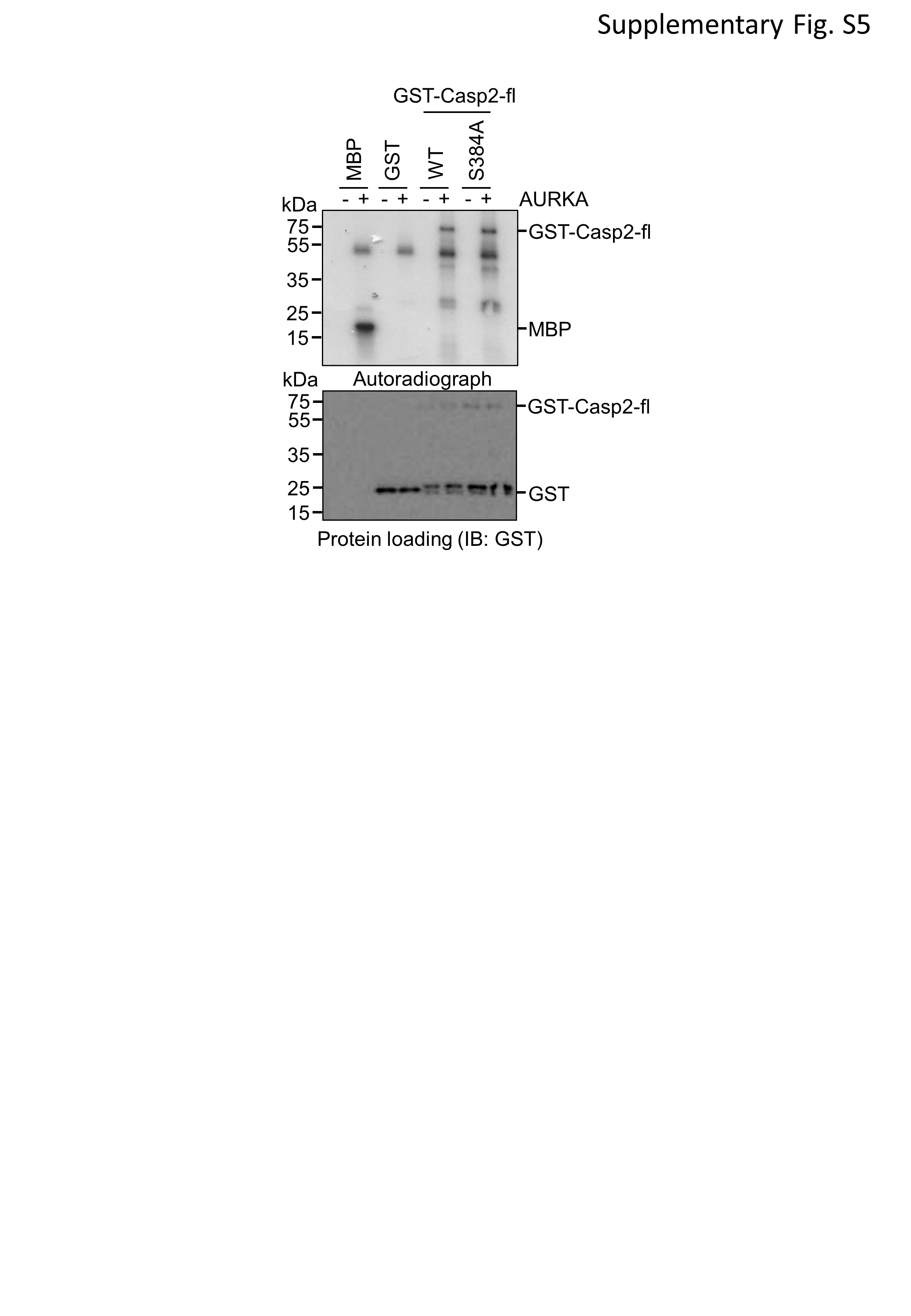

Supplement: Supplementary file 7 — Supplemental Figure 5 [file 41418_2020_604_MOESM7_ESM.tif]

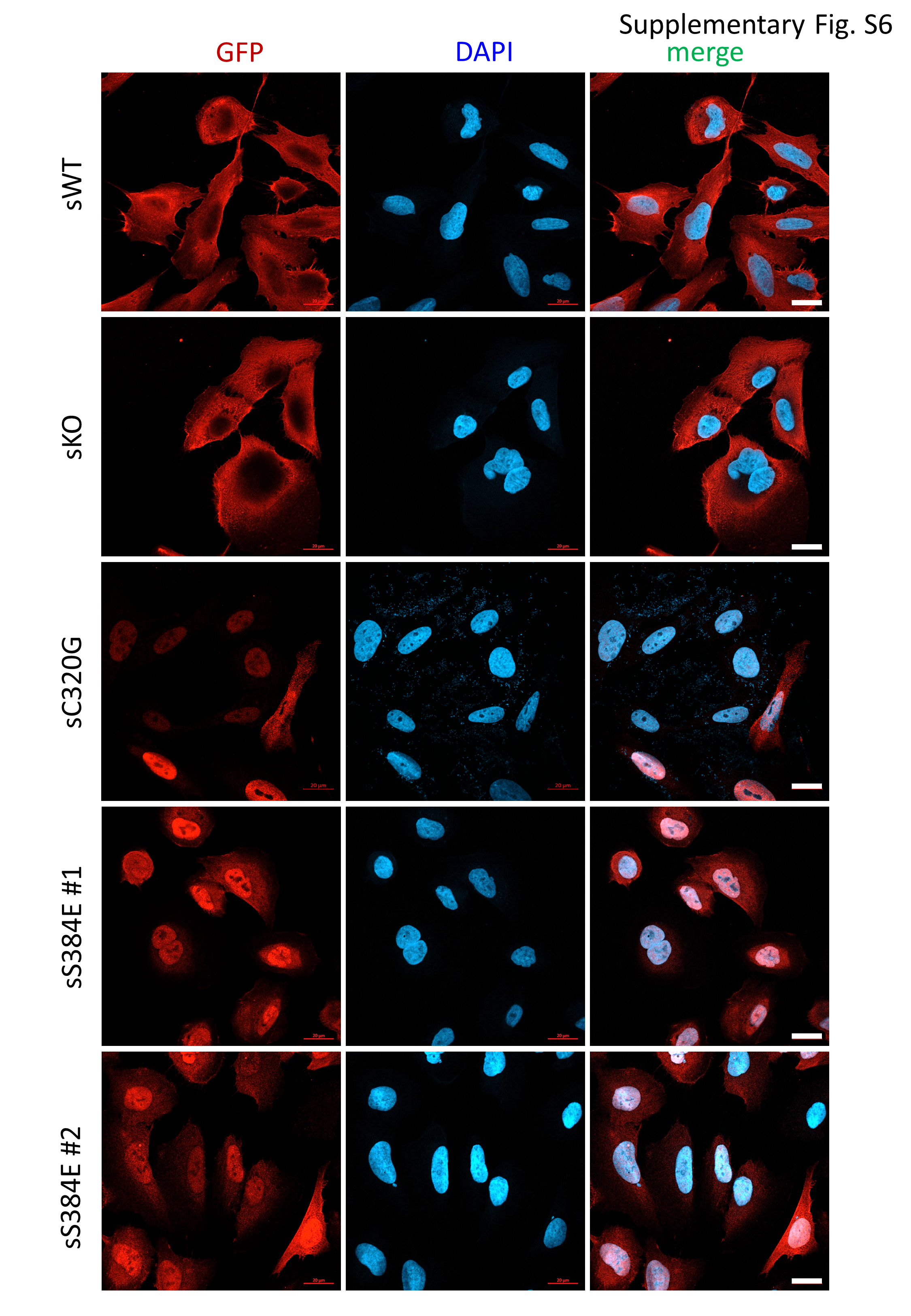

Supplement: Supplementary file 8 — Supplemental Figure 6 [file 41418_2020_604_MOESM8_ESM.tif]

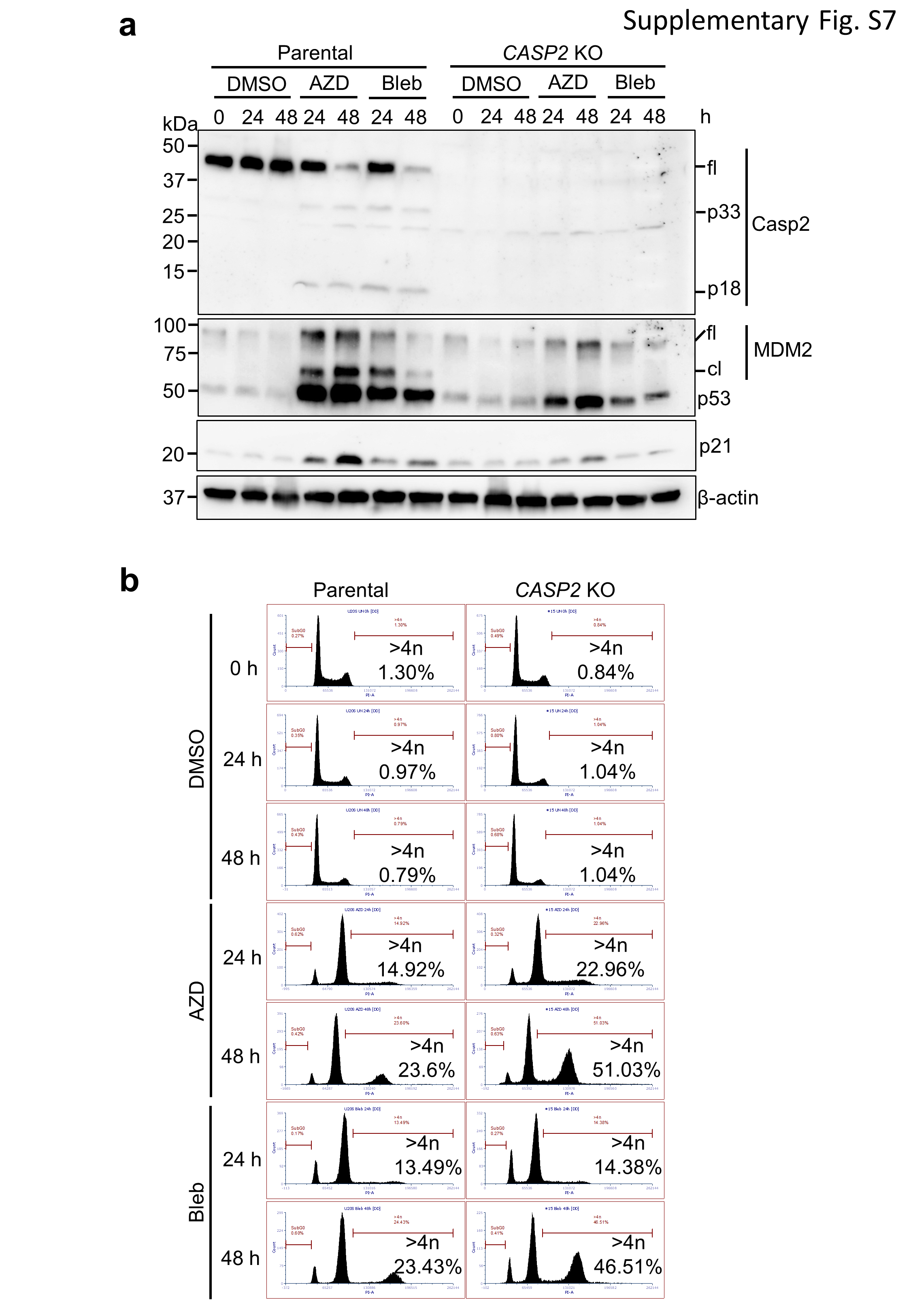

Supplement: Supplementary file 9 — Supplemental Figure 7 [file 41418_2020_604_MOESM9_ESM.tif]

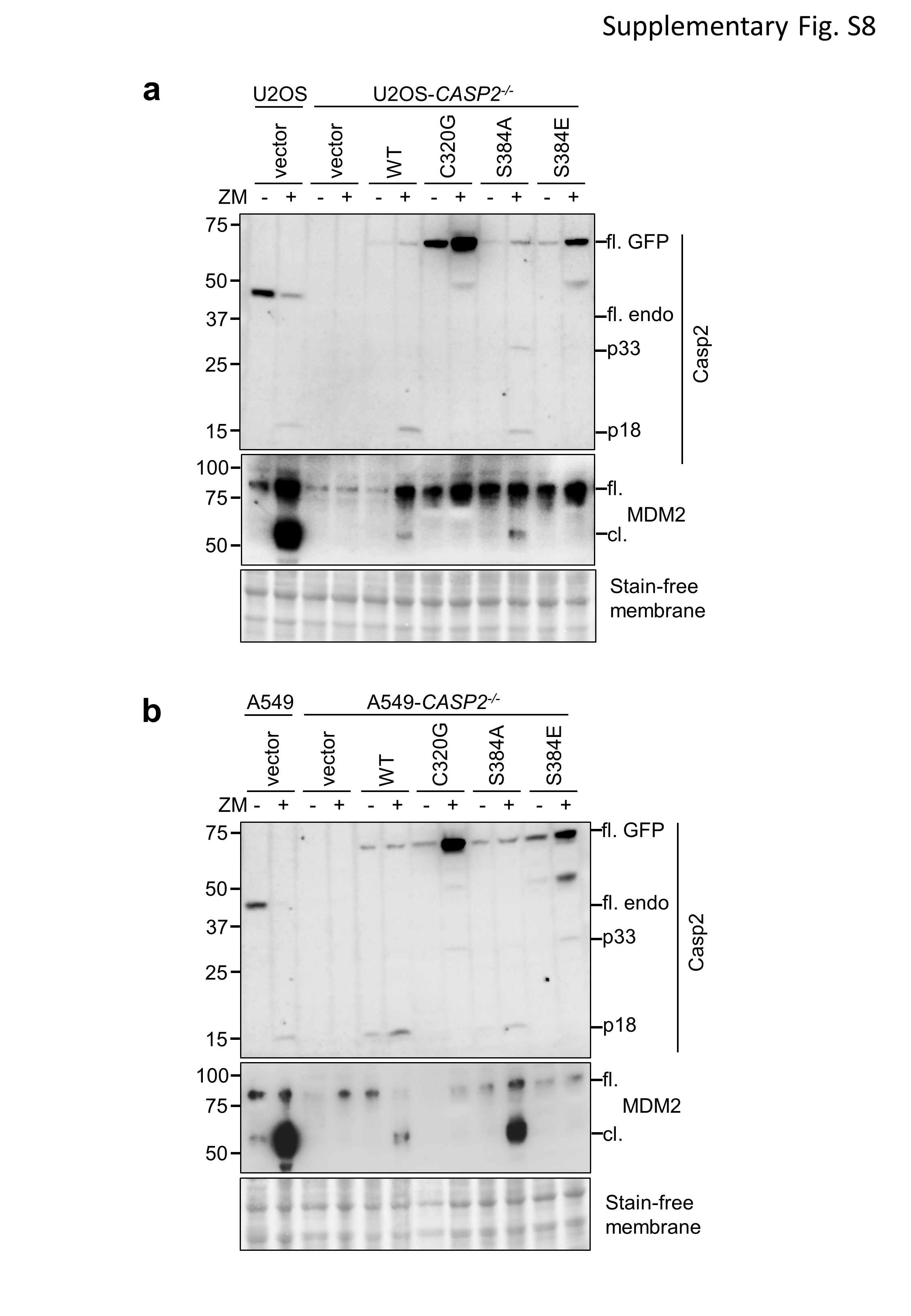

Supplement: Supplementary file 10 — Supplemental Figure 8 [file 41418_2020_604_MOESM10_ESM.tif]

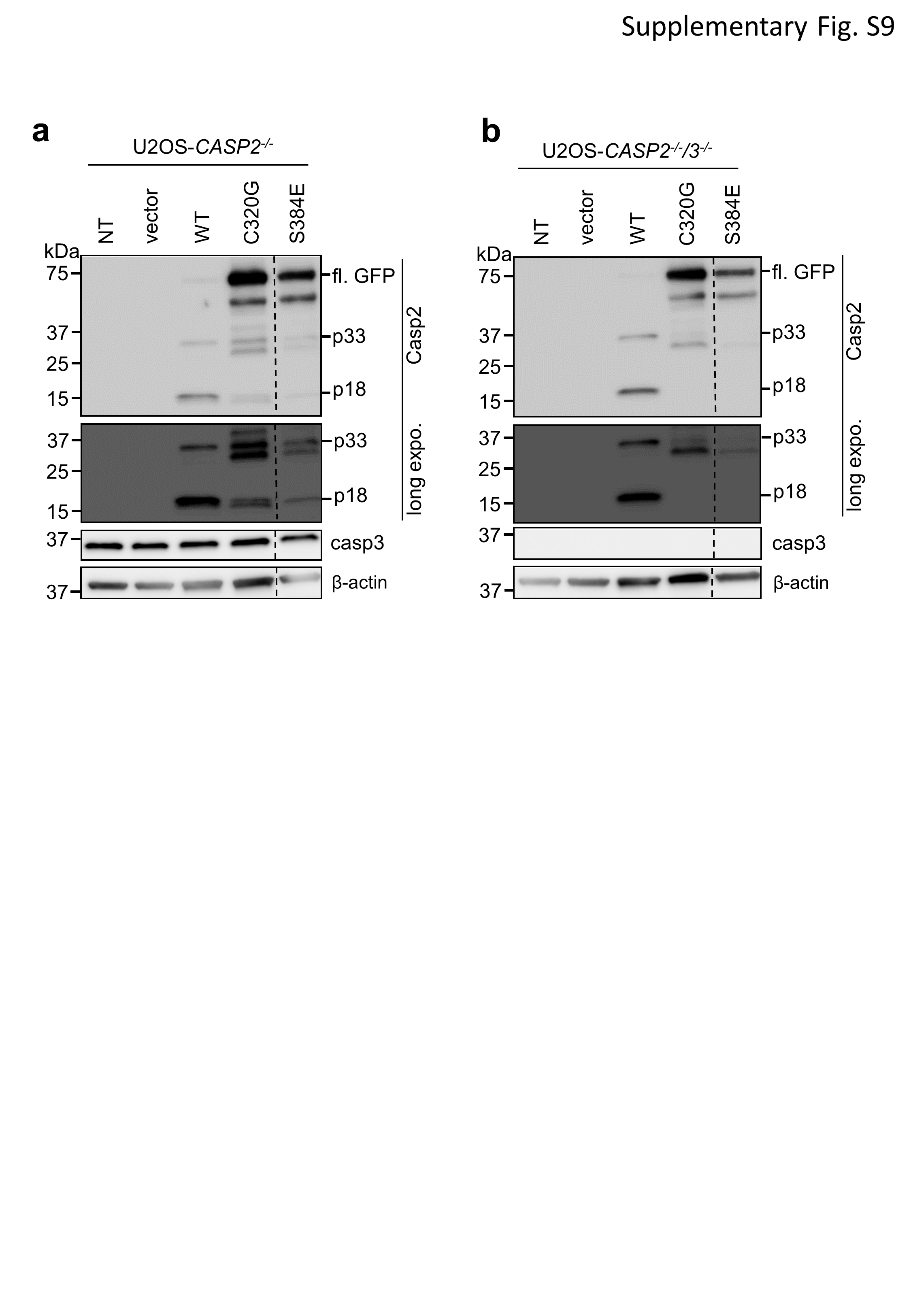

Supplement: Supplementary file 11 — Supplemental Figure 9 [file 41418_2020_604_MOESM11_ESM.tif]
